# Mutational load in carotid body tumor

**DOI:** 10.1186/s12920-019-0483-x

**Published:** 2019-03-13

**Authors:** Anna V. Kudryavtseva, Elena N. Lukyanova, Dmitry V. Kalinin, Andrew R. Zaretsky, Anatoly V. Pokrovsky, Alexander L. Golovyuk, Maria S. Fedorova, Elena A. Pudova, Sergey L. Kharitonov, Vladislav S. Pavlov, Anastasiya A. Kobelyatskaya, Nataliya V. Melnikova, Alexey A. Dmitriev, Andrey P. Polyakov, Boris Y. Alekseev, Marina V. Kiseleva, Andrey D. Kaprin, George S. Krasnov, Anastasiya V. Snezhkina

**Affiliations:** 1 Engelhardt Institute of Molecular Biology, Russian Academy of Sciences, Moscow, Russia; 2 Vishnevsky Institute of Surgery, Ministry of Health of the Russian Federation, Moscow, Russia; 3 National Medical Research Radiological Center, Ministry of Health of the Russian Federation, Moscow, Russia

**Keywords:** Carotid body tumor, Mutational load, Somatic variants, Germline variants, Exome, High-throughput sequencing

## Abstract

**Background:**

Carotid body tumor (CBT) is a rare neoplasm arising from paraganglion located near the bifurcation of the carotid artery. There is great intra-tumor heterogeneity, and CBT development could be associated with both germline and somatic allelic variants. Studies on the molecular genetics of CBT are limited, and the molecular mechanisms of its pathogenesis are not fully understood. This work is focused on the estimation of mutational load (ML) in CBT.

**Methods:**

Using the NextSeq 500 platform, we performed exome sequencing of tumors with matched lymph node tissues and peripheral blood obtained from six patients with CBT. To obtain reliable results in tumors with low ML, we developed and successfully applied a complex approach for the analysis of sequencing data. ML was evaluated as the number of somatic variants per megabase (Mb) of the target regions covered by the Illumina TruSeq Exome Library Prep Kit.

**Results:**

The ML in CBT varied in the range of 0.09–0.28/Mb. Additionally, we identified several pathogenic/likely pathogenic somatic and germline allelic variants across six patients studied (including TP53 variants).

**Conclusions:**

Using the developed approach, we estimated the ML in CBT, which is much lower than in common malignant tumors. Identified variants in known paraganglioma/pheochromocytoma-causative genes and novel genes could be associated with the pathogenesis of CBT. The obtained results expand our knowledge of the mutation process in CBT as well as the biology of tumor development.

## Background

Carotid body tumor (CBT) is the most frequent paraganglioma of the head and neck that arises from carotid glomus [1]. This tumor is highly vascularized and commonly involves carotid artery and cranial nerves. Surgery is a main method for CBT treatment, since radiation therapy and chemotherapy are not very effective.

Tumor development is closely associated with the accumulation of somatic mutations, which may be due to various processes such as endogenous and exogenous DNA damage, defective mechanisms of DNA replication, modification, and repair [2, 3]. These cause the changes in expression profiles of many genes, including activation of oncogenes and inactivation of tumor suppressor genes that lead to alterations in signaling pathways, cellular metabolism, and proliferation [4–14]. Distinct combinations of mutation types (“mutational signatures”) depend on different mutation processes; multiple mutation processes generate jumbled composite signatures. In the study of Alexandrov et al. (2013), more than 21 mutational signatures for 30 cancers were identified, and it has been shown that the prevalence of somatic mutations across human cancer types are different, ranging from approximately 0.04 to 20 somatic mutations per megabase (Mb) [15, 16].

In recent years, immunotherapy has been successfully used in cancer treatments. Notably, the blockade of immunosuppressive checkpoints, such as T-lymphocyte-associated antigen 4 (CTLA4), programmed cell death 1 (PD1) protein, and programmed cell death-ligand 1 (PD-L1), has demonstrated objective clinical responses in various cancers and other malignant neoplasms [17, 18]. Inhibition of both CTLA4 and PD1/PD-L1 reactivates lymphocytes against tumor-expressing neoantigens. They result from different mutations in tumor cells, and the potential of neoantigen formation is correlated with mutational load (ML) [19]. Melanoma and lung cancer, which are characterized by high MLs, were demonstrated to have clinical benefits from the immunotherapy with antibodies targeting CTLA4 and PD1 [20, 21]. Responses to immune checkpoint blockade therapy have been described in patients with colorectal cancer characterized by microsatellite instability (MSI), which is accompanied in most cases with high ML, resulting from defects in mismatch-repair pathways [22]. High ML, MSI (surrogate marker of high ML), and neoantigen production have been demonstrated to be promising markers of sensitivity to immune checkpoint blockade for several tumors [23–25]. However, these criteria also exhibited inconsistent patterns in patients with ovarian and urothelial cancer as well as those with glioblastoma; therefore, their potential use as prognostic factors requires further studies [26–28]. Additionally, intestinal microbiota, expression of PD-L1 in tumor cells, and tumor-infiltrating lymphocytes (TILs) were also determined to have a predictive role in immunotherapeutic responses [29–33].

In this study, we estimated the ML in CBT. We performed exome sequencing of tumors with matched lymph node tissues and peripheral blood derived from six patients with CBT. Additionally, a number of pathogenic/likely pathogenic somatic and germline variants were identified.

## Methods

### Patients and samples

Formalin-fixed paraffin-embedded (FFPE) tumor and lymph node tissues as well as peripheral blood from six patients with CBT were collected from Vishnevsky Institute of Surgery, Ministry of Health of the Russian Federation for exome sequencing. We also used a collection of 52 CBTs (exome sequencing data, available in the NCBI Sequence Read Archive [SRA] under accession number PRJNA411769) from a previous study [34]. All patients provided written informed consent. Clinicopathologic characteristics of the patients with CBT are presented in Table 1. The study was approved by the ethics committee from Vishnevsky Institute of Surgery and performed according to the Declaration of Helsinki (1964).

**Table 1:** Clinicopathologic characteristics of the patients with CBT

Patient	Gender	Age (years)	Family history of paragangliomas	Metastasis (lymph node/distance)	Multifocal growth	Comments
Pat100	Female	35	N/A	No	No	–
Pat101	Female	46	N/A	No	No	Tumor recurrence was diagnosed in a year after surgery*
Pat102	Female	31	N/A	No	No	–
Pat103	Male	57	N/A	No	No	–
Pat104	Female	58	N/A	No	Yes	Carotid body tumor and vagal paraganglioma were diagnosed*
Pat105	Female	67	N/A	No	No	–

* - In the study, only primary CBT from Pat101 and CBT (not vagal paraganglioma) from Pat104 were analyzed

### Exome sequencing

DNA was extracted from blood cells using a MagNA Pure Compact Nucleic Acid Isolation Kit I (Roche, Switzerland) on a MagNA Pure Compact Instrument (Roche); DNA from tumor and lymph node tissues was isolated with High Pure FFPET DNA Isolation Kit (Roche). DNA (100 ng per sample) was sheared to 150 bp using Covaris S 220 System (Thermo Fisher Scientific, USA) and was then subjected to library preparation with TruSeq Exome Library Prep Kit (Illumina, USA) according to manufacturer’s instructions. The exome sequencing was performed on a NextSeq 500 System (Illumina) with paired-end reads. Read length was 76 bp for tumor and lymph node tissues and 151 bp – for blood. The obtained coverage was at least 300×. Raw sequencing data have been deposited at the NCBI SRA under accession number PRJNA476932.

Raw reads were trimmed and adapter sequences were removed with Trimmomatic [35]. We aligned 100 K randomly selected reads to bacterial genomes (NCBI, all bacterial genomes submitted up to 2014) with BWA [36] in order to evaluate contamination levels. All the samples demonstrated no greater than 0.1% bacterial DNA ratios. Next, reads were mapped to the reference human genome GRCh37.75 (Ensembl) with BWA. The derived BAM files were processed with picard-tools (reordered, supplied with group names, and duplicated reads were marked). Then, we performed base quality score recalibration (BQSR) using GATK4 (version 4.0.8.1) and dbSNP (common variants, 2015-06-05). To call somatic variants, two algorithms, VarScan [37] and Mutect2 [38], were used.

First, we applied VarScan to reveal somatic variants in paired (‘tumor *versus* normal’) mode. We merged BAMs for blood and lymph nodes per each patient and submit pileups (bcftools) from these BAMs to VarScan. Reads with mapping quality lower than 20 and the bases with base calling quality lower than 20 were filtered out. Only regions with 20x or higher coverage (for both tumor and ‘merged’ norm) were included in the analysis.

Second, we used Mutect2 (from GATK 4.0.8.1) to identify somatic variants, SNVs and indels. Before calling somatic variants, we ran Mutect2 in ‘tumor-only’ mode with all the 12 normal samples (lymph nodes and blood) to create a panel of norms (PoN). Next, we merged BAMs for blood and lymph nodes per each patient and used these BAMs along with PoN to call somatic variants with Mutect2 in ‘tumor *versus* normal’ mode. The derived VCFs were analyzed with GATK FilterMutectCalls, and only passed somatic variants were included in the further analysis. We have decided to also include in the analysis clustered events, which are filtered out with FilterMutectCalls by default. Additionally, we called variants in artificial comparisons ‘lymph node (FFPE) *versus* blood’, ‘tumor (FFPE) *versus* blood’, ‘blood *versus* tumor (FFPE)’, etc.

The derived list of somatic variants was annotated using Annovar [39]. We included allele population frequency databases (gnomAD, 1000 Genomes Project, Kaviar, ESP 6500, and ExAC), public variant databases (dbSNP, ClinVar, and COSMIC), phastCons containing conservation data for vertebrates, primates, and placental mammals [40], and InterPro to analyze the localizations of variants in protein domains [41]. Additionally, prediction tools such as SIFT [42], PolyPhen2 [43], MutationTaster [44], LRT [45], InterVar [46], PROVEAN [47], M-CAP [48], MetaSVM, and MetaLR [49] were used to assess the pathogenicity of the variants. Variants were considered to be likely pathogenic if they were predicted as deleterious by at least three algorithms. However, in most cases the majority of algorithms gave consistent results.

We excluded variants with population frequency greater than 1%. Worth noting, the overall number of such variants comprised only 0–7% of all the exonic somatic variants (variants in gene coding regions) passed after analysis with FilterMutectCalls. Additionally, the list of somatic variants was filtered according to the minimal read coverage threshold (min 20 reads for a merged norm and min 10 reads for the tumor sample).

## Results

### Mutational load in CBT

First, we should mention once more that we used blood and FFPE samples taken from tumor and lymph node tissues. The accurate detection of variants in FFPE samples is often problematic because of DNA fragmentation and the occurrence of sequence artifacts resulted from fixation of tissues in formaldehyde [50]. To evaluate the effect of FFPE artifacts on the results, we compared ‘lymph node (FFPE) *versus* blood’ and revealed a great number of variants (hundreds) that was almost equal to the number of somatic variants found in the ‘tumor (FFPE) *versus* blood’ comparison (including variant with low alternative allele (AF) frequency). In contrast, when comparing either ‘blood *versus* tumor (FFPE)’ or ‘blood *versus* lymph node (FFPE)’, a very moderate number of variants (dozens) was revealed. This pronounced trend was observed for all six patients. It suggests that the most of variants identified in FFPE samples may be formalin-induced DNA artifacts. However, there were a variety of SNVs (A > T, C > A, G > A, A > G, etc.); and only a moderate bias towards the typical FFPE-induced transition, C > T, was observed. About 25% of all SNVs were represented with C > * substitutions, and approximately 40–60% of them were C > T transitions (10–15% of all SNVs).

When ‘tumor *versus* lymph node’ was compared, e.g. two FFPE samples, we derived about 1.3–1.5-fold lower amount of somatic variants relatively ‘tumor (FFPE) *versus* blood’ comparison, because read coverage for FFPE lymph node samples was 2-fold greater (on the average) than the coverage for blood samples. Additionally, this may suggest slight co-occurrence of formalin-induced variants, which are partially ‘subtracted’ when comparing two FFPE samples.

Among Mutect results, there are several false-positive somatic variants with low AF (5–10%) but high coverage (10–30 reads for alternative allele; the total coverage was at least 300x) observed in tumor, lymph node and blood samples. These variants obviously are neither germinal (too low AF), nor somatic (they are present in norms and tumors), nor FFPE artifacts (they are present in blood samples). Remarkably, 20–40% of reads that support the alternative alleles (AA) have low base quality score (before and after BQSR). This strongly suggests the presence of context-dependent sequencing errors that are not eliminated with BQSR procedure and are not sufficiently addressed by a variant calling algorithm (either by Mutect2 or VarScan). Indeed, we observed many of these variants in polyN-tracts (especially polyG), e.g. GGGT>GGGG, CCCCG>CCCCC, susceptible for Illumina NextSeq-specific sequencing errors.

To eliminate such artifacts, we applied three approaches. First, we used strand bias filter (StrandOddsRatio annotation provided with GATK Mutect2 or HaplotypeCaller). The presence of reads bearing a variant on only one strand indicates a false-positive. However, this option alone does not allow eliminating artifacts when they come from both strands (e.g. GGGTGGG>GGGGGGG). Second, we filtered out variants with abnormal distribution of base quality scores across the reads (GATK BaseQualityRankSumTest annotation). In other words, we excluded variants that were abnormally supported with too many reads with low base quality score at a current position, even if there were also many reads with high base quality score at this position. Third, we manually filtered out variants that were observed after/before four or more identical nucleotides (mainly, C/G). In most cases, when we are calling somatic variants on various types of cancer, their number may significantly exceed the ratio of the described false-positives. In contrast, when analyzing tumors with a low ML, this issue becomes especially important.

**Fig. 1:**
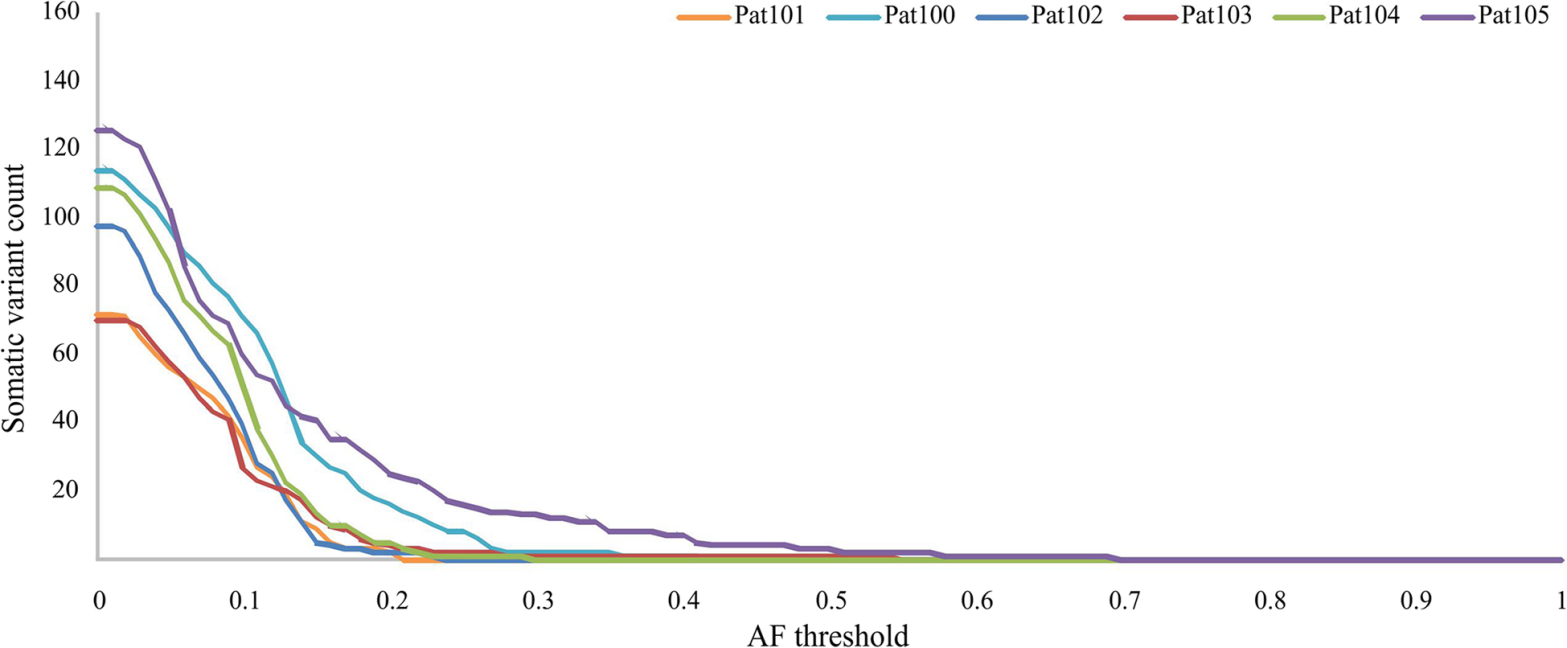
Somatic variant count depending on minimum AF threshold

Totally, we found 70–130 potentially somatic variants in each patient comparing ‘tumor (FFPE) *versus* matched lymph node (FFPE) and blood + PoN’. Most of these variants have low AF (Fig. 1). Only 5–20% of variants passed threshold of AF > 15%.

**Fig. 2:**
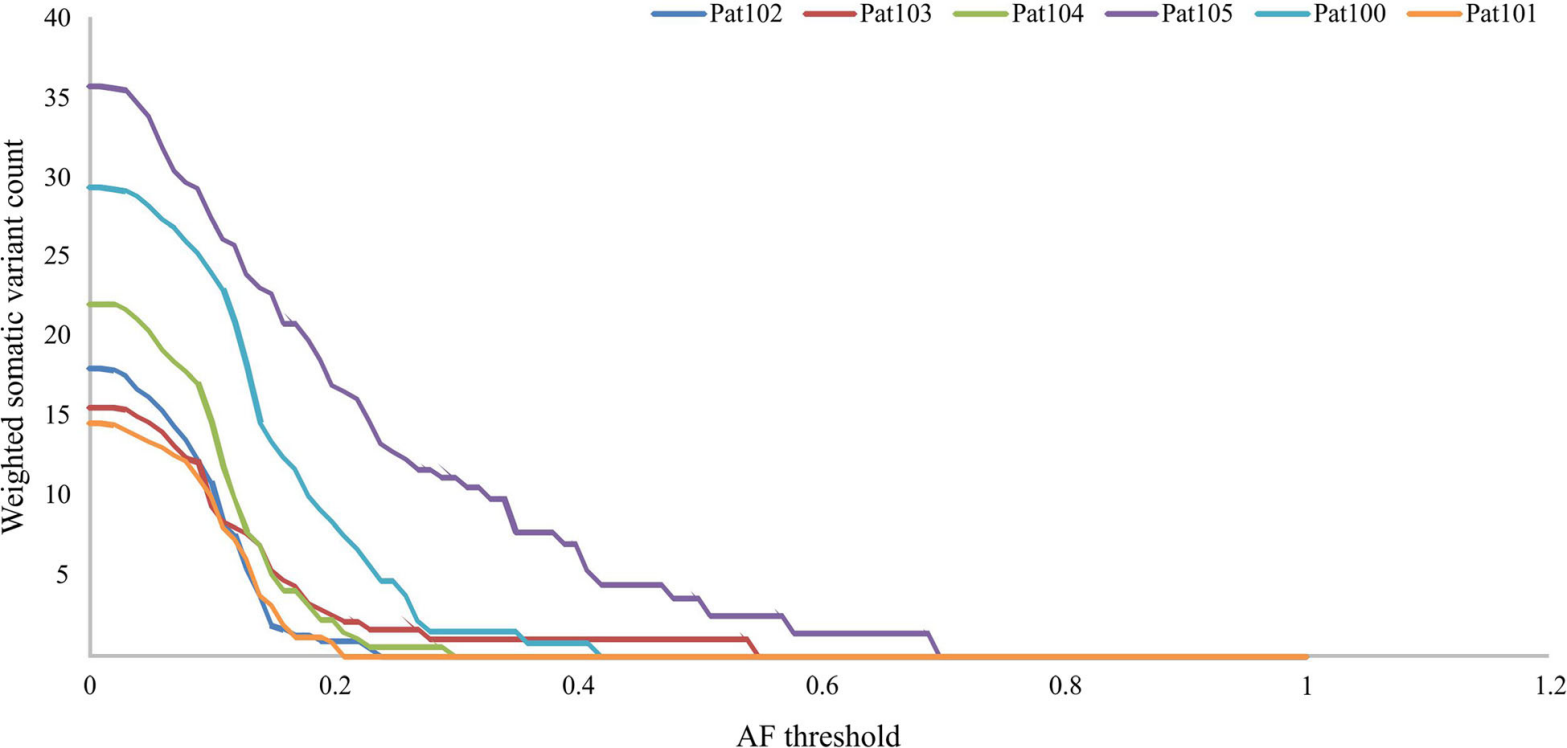
Weighted somatic variant count depending on minimum AF threshold

When calculating the total mutational load, it is incorrect to consider a somatic variant that is observed only in a fraction of the tumor cells (such variants are featured with low AF) as a “whole one”. Otherwise, the higher sequencing coverage, the more we can find variants with very low AF values, and the higher calculated mutational load will be. The weight of such somatic variants should be adjusted for AF. Hence, we re-estimated the number of variants “in terms of heterozygous ones” as the sum of all AFs multiplied by 2 (Fig. 2, weighted somatic variants count).

As can be seen from Fig. 2, two of six patients demonstrated an elevated number of potentially somatic variants. These differences are much more pronounced when an elevated AF threshold is set (AF > 0.2, e.g. > 40% of cells are for heterozygous variant). Nevertheless, it is worth noting that the initial number of tumor cells in the tumor samples did not reach 100%, and it could vary from 70 to 90%.

To finally assess the weighted mutational load (wML) in CBT, we should reasonably pick up the AF threshold. For most patients, a significant reduce in the number of variants occurs in the region of AF = 10–15%. Therefore, wML may be estimated as 4–12 variants per genome or 0.09–0.28 variants per megabase taking into account the fact that we have used TruSeq Exome Library Prep Kit (the total length of target regions is 43 Mb). It should be noted that the evaluation of ML in such cases is close to the limit of sensitivity/specificity of the method.

**Fig. 3:**
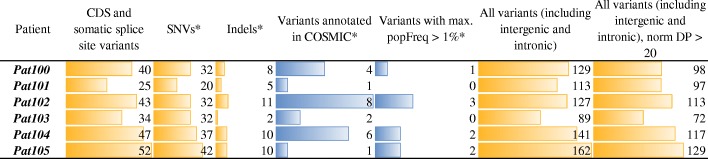
A number of somatic variants located in coding regions or splice sites (SNVs and indels) across six patients. The threshold of minimal number of high-quality reads supporting an alternate allele was set as 3. CDS – coding sequence; DP – depth (the sum of alt+ref read coverage) * - The number of SNVs, indels, and annotated variants are calculated for the subset of CDS/splice variants

Considering the structure of a list of potential somatic variants, 25–73 variants (SNVs and indels) are located either in coding regions or splice sites and are supported with at least three reads corresponding to the alternative allele (Fig. 3). Remarkably, 2–18% of these ones have already been annotated in COSMIC databases, and only 2–7% of variants have maximal population frequency (across multiple databases) greater than 1% (before filtering). The total number of all variants (including UTRs, intronic, and intergenic) was 2-fold greater on the average than the number of variants in the coding regions.

### Pathogenic and likely pathogenic somatic variants

**Fig. 4:**
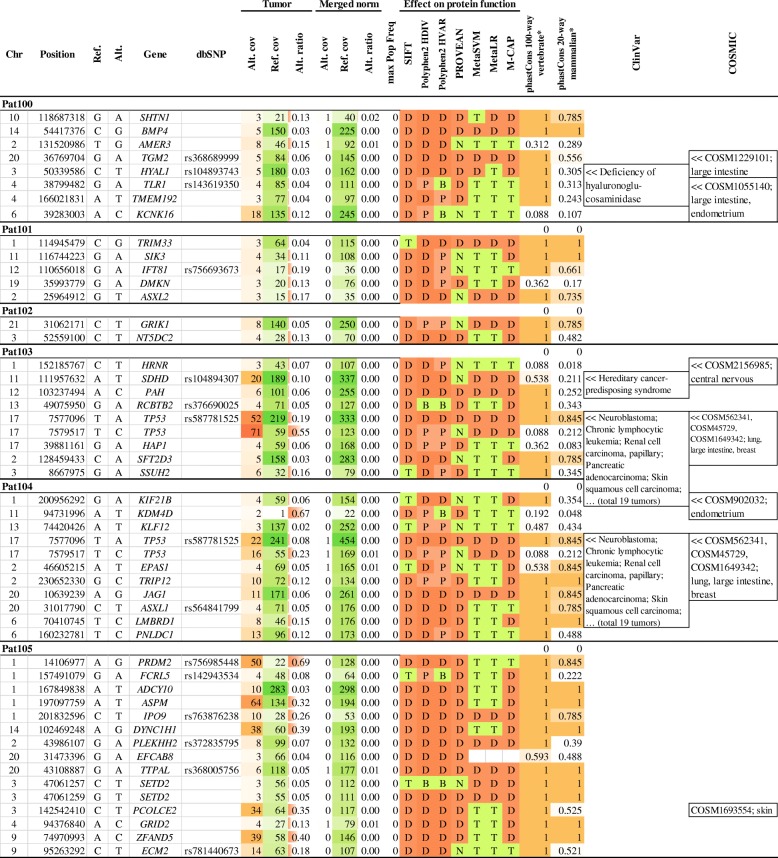
Pathogenic/likely pathogenic somatic variants in six samples of carotid body tumor

Across six patients, we revealed 50 likely pathogenic variants, and among them several potential driver variants were observed (Fig. 4). In two patients (Pat103 and Pat104) we found two co-occurred variants in *TP53* gene, NM_000546.5: c.842A > T, p.Asp281Val (chr17: 7,577,096, rs587781525) and NM_000546.5: c.A170A > G, p. Asp57Gly (chr17: 7,579,517). The first variant was described in dbSNP as both germline and somatic one, and has a pathogenic clinical significance according to the ClinVar database. The germline variant was associated with hereditary cancer-predisposing syndrome; the somatic variant has been found in many neoplasms, including neuroendocrine tumors (neuroblastoma and glioblastoma). Variant NM_000546.5: c.A170A > G, p. Asp57Gly (chr17: 7579517) was not previously described in databases and in the literature. In our study, this variant was found with high AF (0.55 and 0.23 for Pat103 and Pat104, respectively) and was classified to be likely pathogenic by most prediction tools. These two variants can be associated with biallelic inactivation of *TP53* gene and are involved in the pathogenesis of CBT.

Potential driver variant NM_003002: c.A1A > T, p. Met1Leu (chr11: 111,957,632, rs104894307) in *SDHD* gene was revealed for Pat103. It was annotated in dbSNP and ClinVar databases as germline pathogenic variant associated with hereditary cancer-predisposing syndrome and pheochromocytomas/paragangliomas. This somatic variant was also described as pathogenic in sporadic paraganglioma [51].

We also observed somatic variants in several known cancer-associated genes (for example, *JAG1, PRDM2, PRDM8, SETD2, ASPM, ZIC, GRIK1,* etc.), which may be important for cell growth and proliferation. They did not overlap between the patients. These variants have low AF values (5–10%) occurring in low fraction of cells (10–20%), but they may represent driver events. This demonstrates genetic intra-tumor heterogeneity of CBT. Apparently, they occurred after tumor onset and initial progression. Having received these variants, such cells could gain an advantage in their clonal expansion.

### Pathogenic and likely pathogenic germline variants in causative genes

**Fig. 5:**
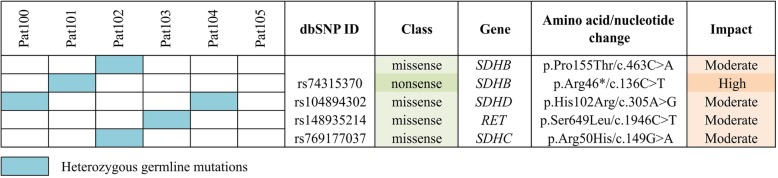
Distribution of pathogenic/likely pathogenic germline variants across six carotid body tumor samples

We analyzed germline variants in 42 genes (*VHL, SDHA, SDHB, SDHC, SDHD, NF1, RET, HRAS, KRAS, EPAS1 (HIF2A), ATRX, CSDE1, BRAF, FGFR1, FGFR2, FGFR3, FGFR4, FGFRL1, SETD2, ARNT, TP53, TP53BP1, TP53BP2, TP53I13, KMT2D, BAP1, IDH1, IDH2, SDHAF1, SDHAP2, FH, EGLN1, MDH2, TMEM127, MAX, KIF1B, MEN1, GDNF, GNAS, CDKN2A, BRCA1,* and *BRCA2*) reported previously to be involved in the development of paragangliomas/pheochromocytomas [34, 52]. Three pathogenic and two likely pathogenic germline variants were found across six patients with CBT according to the predicted algorithms and public databases (Table 2, Fig. 5). These variants were characterized by high conservation scores (PhastCons) and had an allele frequency less than 0.01% in 1000 Genomes Project, ESP 6500, and ExAC databases.

**Table 2:** Pathogenic/likely pathogenic germline variants in six samples of carotid body tumor

Gene	dbSNP	GenBank	Coordinate	Nucleotide change	Amino acid change	Genotype	Predictions				Clinical significance (dbSNP/ClinVar)
							SIFT	PolyPhen2	MutationTaster	LRT	
*SDHB*	–	NM_003000.2	chr1: 17,354,321	c.463C > A	p.Pro155Thr	Het	Deleterious (0)	Probably damaging (1)	Disease-causing (*p*-values = 1)	Neutral (0)	N/A
*SDHB*	rs74315370	NM_003000.2	chr1: 17,371,320	c.136C > T	p.Arg46*	Het	Deleterious (0)	Benign (0)	Disease-causing (*p*-values = 1)	Neutral (0)	Pathogenic
*SDHD*	rs104894302	NM_003002.3	chr11: 111,959,726	c.305A > G	p.His102Arg	Het	Deleterious (0)	Probably damaging (0.99)	Disease-causing (*p*-values = 1)	Neutral (0)	Pathogenic
*RET*	rs148935214	NM_020975.4	chr10:43,609,994	c.1946C > T	p.Ser649Leu	Het	Deleterious (0)	Probably damaging (1)	Disease-causing (*p*-values = 1)	Neutral (0)	Pathogenic
*SDHC*	rs769177037	NM_003001.3	chr1: 161,298,257	c.149G > A	p.Arg50His	Het	Deleterious (0)	Probably damaging (1)	Not disease-causing (*p*-values = 0)	Neutral (0)	Uncertain significance

## Discussion

The frequency of somatic mutations and neoantigen production correlates with responses to immunotherapy. In high mutated cancer, the ML was considered as a prognostic factor of prolonged survival with immune checkpoint inhibitors [20, 21]. However, this association has not been observed for tumors with low ML. In the present work, we estimated the ML in CBT. CBT belongs to rare neoplasms, and it is difficult to collect a representative set of CBT samples. Moreover, matched morphological normal tissues (“conventional norm”) are unavailable due to the tumor localization. We collected and performed exome sequencing of tumor samples with matched lymph node tissues and peripheral blood from six patients with CBT. This revealed actual somatic variants and allowed estimating the ML in the six samples studied. CBT carried low ML (0.09–0.28/Mb) compared to other common cancers [15]. Even though high ML is a factor for immunotherapy, it also generally indicates high aggressiveness of the tumors and correlates with increased genetic instability and poor prognosis [53–56]. CBT is usually a slow-growing tumor, and in 10% of cases, it can become aggressive and metastatic [57]. Thus, low ML in CBT is possibly associated with its non-aggressive behavior and probably indicates inefficiency of immune checkpoint blockade therapy.

Paragangliomas/pheochromocytomas can be caused by germline and somatic variants in at least 42 known genes [52]. Potential driver somatic variants were found in *SDHD* (Pat103) and *TP53* (Pat103 and Pat104) genes. Likely pathogenic variants were revealed in *EPAS1* (*HIF2A*) (Pat104) and *SETD2* (Pat105) genes, and no somatic variants were observed in other known genes. However, several pathogenic and likely pathogenic germline variants in *SDHB, SDHC, SDHD*, and *RET* genes were detected. A majority of them were identified in *SDHx* genes that are often associated with hereditary pheochromocytomas/paragangliomas [58]. Two variants were observed in *SDHB* gene in different patients (Pat101 and Pat102): a novel missense variant NM_003000.2: c.463C > A, p.Pro155Thr (chr1: 17,354,321) and a nonsense high-impact variant NM_003000.2: c.136C > T, p.Arg46* (chr1: 17,371,320, rs74315370). The latter was described in the dbSNP and ClinVar databases as a pathogenic germline variant associated with hereditary cancer predisposition syndrome, paragangliomas/pheochromocytomas, and gastrointestinal stromal tumor [59–62]. Notably, this variant has been reported in patients with aggressive extra-adrenal paraganglioma in the chest and CBT and has been considered as a high-risk factor for malignancy or recurrence of paragangliomas/pheochromocytomas [63–66]. Indeed, the patient tested (Pat101) was characterized by the tumor recurrence that is one of the features indicating aggressive phenotype of CBT. The variant NM_003001.3: c.149G > A, p.Arg50His (chr1: 161,298,257, rs769177037) in *SDHC* was also found in Pat102. It was described in dbSNP as a variant of uncertain clinical significance. Germline variants in *SDHC* are more rarely associated with the development of  paragangliomas/pheochromocytomas than variants in *SDHB* or *SDHD* [58]. It should be noted that in Pat102 we observed two somatic likely pathogenic variants in *GRIK1* and *NT5DC2* genes. Therefore, according to our previous data, the formation of CBT can be probably caused by the cumulative effect of several highly or not highly pathogenic variants [34]. In this particular case, it seems that the main driver is the pathogenic germline variant in *SDHB* gene.

The germline variant NM_003002.3: c.305A > G, p.His102Arg (chr11: 111,959,726, rs104894302) in *SDHD* was identified in two patients – Pat100 and Pat104. This variant is found in dbSNP and ClinVar databases as a pathogenic germline variant associated with hereditary cancer-predisposing syndrome, paragangliomas/pheochromocytomas, gastric stromal sarcoma, and Cowden syndrome 3 [67, 68]. This variant has been detected in malignant CBT [69]. Data on the aggressive behavior of the tumor in tested patients have not been reported; one patient (Pat104) was characterized by multiple tumors (vagal paraganglioma and CBT) with multifocal growth. In this patient (Pat104), we also found pathogenic and likely pathogenic somatic variants in *TP53* gene.

One patient (Pat103) carried the germline variant NM_020975.4: c.1946C > T, p.Ser649Leu (chr10: 43,609,994, rs148935214) in the proto-oncogene *RET*. It was deposited to dbSNP from the gnomAD database as a germline variant. In ClinVar, another allele was reported with conflicting interpretations of pathogenicity found in hereditary cancer-predisposing syndrome (uncertain significance) and multiple endocrine neoplasia (MEN) type 2 (uncertain significance/likely benign) characterized by medullary thyroid carcinoma, pheochromocytomas, and hyperparathyroidism [70–72]. In this patient, we also identified pathogenic and likely pathogenic somatic variants in *TP53* gene (NM_000546.5: c.842A > T, p.Asp281Val (chr17: 7,577,096, rs587781525) and NM_000546.5: c.A170A > G, p. Asp57Gly (chr17: 7,579,517) that are the same in Pat104. These variants can be potential driver ones.

Interestingly, we did not reveal any pathogenic germline variants in known paraganglioma/pheochromocytoma-causative genes in the patient Pat105, which is characterized with the greatest number of somatic variants and the highest ML. However, this patient was characterized by at least two somatic variants in CBT in one known CBT-causative gene – *SETD2*. This gene encodes for histone methyltransferase, an epigenetic modifier with tumor suppressor functionality [73]. Mutations in *SETD2* are found in many tumors, including neoplasms of the central nervous system [74].

In the previous work, we performed exome sequencing of 52 archival FFPE samples of CBT [34]. Peripheral blood or other normal tissues were unavailable; therefore, germline variants were excluded with strong filtering using the 1000 Genomes Project and ExAC databases. We estimated the ML as the number of potentially somatic deleterious variants per megabase of coding regions. However, we derived an obviously elevated ML: the average ML was 6–8 variants per Mb. Therefore, such approach does not allow efficient elimination of germline variants. Indeed, this method excludes 96–98% or more germline variants, but the remaining 2–3% of the germline variants may significantly outnumber somatic ones. In this study, we filtered the pool of somatic variant candidates from a previous work (52 patients) using exome sequencing data on blood and lymph nodes derived in the present work (6 patients). This resulted in at least 2-fold reduction of the estimated ML, but this value was still excessively high. Thus, the use of matched normal tissues is necessary to be able to accurately estimate ML.

Recently, Roche (Switzerland) announced the AVENIO ctDNA Analysis Kits for personalized oncology assays [75, 76]. The AVENIO ctDNA Surveillance Kit targets frequently mutated regions across 197 genes and has been optimized for monitoring of ML in lung and colorectal cancers. This kit contains the main genes that are associated with lung, colorectal, breast, gastric, prostate, ovarian, thyroid, and pancreatic cancers, as well as glioma and melanoma according to the U.S. National Comprehensive Cancer Network (NCCN) Guidelines (https://www.nccn.org/). The kit did not include genes that have been shown to be involved in the pathogenesis of paragangliomas and pheochromocytomas, except *TP53*, *BRCA1*, and *BRCA2*. A panel of genes accurately reflecting the ML in CBT is also unknown. Moreover, the kit and the appropriate analysis software focus on quantitating ML basing on ctDNA sequencing, and this approach is more acceptable for malignant tumors with a high frequency of metastases, while CBT is primarily a slow-growing tumor with indeterminate potential of malignancy. Thus, whole exome sequencing, which was used in the study, is currently the only method for estimating the ML in CBT.

## Conclusion

The ML varied in the range of 0.09–0.28/Mb in the analyzed cohort of patients with CBT (six individuals). Several pathogenic/likely pathogenic somatic and germline allelic variants in both known paraganglioma/pheochromocytoma-causative genes and novel ones were identified. These results improve the understanding of CBT pathogenesis.

## Abbreviations

AA: Alternative allele
AF: Allele frequency
CBT: Carotid body tumor
ML: Mutational load
